# In Adjuvant-Induced Arthritic Rats, Acupuncture Analgesic Effects Are Histamine Dependent: Potential Reasons for Acupoint Preference in Clinical Practice

**DOI:** 10.1155/2012/810512

**Published:** 2012-11-05

**Authors:** Meng Huang, Di Zhang, Zhe-yan Sa, Ying-yuan Xie, Chen-li Gu, Guang-hong Ding

**Affiliations:** ^1^Department of Mechanics and Engineering Science, Fudan University, Shanghai 200433, China; ^2^Shanghai Research Center for Acupuncture and Meridians, Shanghai 201203, China; ^3^College of Acupuncture and Moxibustion, Shanghai University of Traditional Chinese Medicine, Shanghai 201203, China; ^4^Medical School, Fudan University, Shanghai 200433, China

## Abstract

This study investigated whether immediate acupuncture effects in the acupoint are histamine dependent. Both histamine injection and manual acupuncture stimulation increased the pain threshold (PT) after treatment compared with the model group (*P* < 0.01), producing an analgesic effect. After pretreatment with clemastine, an H1 receptor antagonist and an antipruritic, the increase in the animals' pain threshold after acupuncture was suppressed compared with the Acu group (*P* < 0.01); however, there was no interference with the acupuncture-induced degranulation of mast cells. Pretreatment with disodium cromolyn did not suppress the increase in PT induced by the histamine injection at Zusanli (ST-36). We conclude that in adjuvant-induced arthritic rats, acupuncture analgesic effects are histamine dependent, and this histamine dependence determines the acupoint preference of acupoints away from the target site in acupuncture practice.

## 1. Introduction

During the last two decades, research on acupuncture has determined that acupuncture is based on relationships between structures and functions that have been studied under physiological conditions. Regarding the initiation of local acupoint effects after acupuncture, the degranulation of mast cells was found to be related to acupuncture analgesia in adjuvant-induced arthritic rats [1]. Moreover, these analgesic effects depend on the transduction of neural signals above the acupunctured acupoint [2]. However, the characteristics of this neural activation induced by mast cell degranulation after acupuncture remain unclear. In this study, we tested the histamine dependence of acupuncture analgesia and presented a hypothesis for the mechanism of acupoint preference in acupuncture analgesia clinical practice.

## 2. Methods

### 2.1. Animals

The present study was performed in accordance with the guidelines of the Animal Care and Use Committee of Shanghai Research Center for Acupuncture and Meridians. Male Sprague-Dawley (SD) rats (150 ± 20 g), from the Shanghai Experimental Animal Center of the Chinese Academy of Science, were housed in cages with a temperature-controlled environment (22–25°C) and a 12/12-hour light/dark cycle. Food and water were made available ad lib. All animals were handled with care to prevent infection and to minimize stress. All behavioral experiments were performed between 9 am and 4 pm. For each experimental group, animals were chosen randomly. 

### 2.2. Adjuvant Arthritis Model

To achieve the adjuvant arthritis model, rats under anesthesia (10% chloral hydrate 0.4 mL/100 g i.p.) were injected with 0.05 mL of Complete Freund's Adjuvant (Sigma-Aldrich) in the left ankle joint. On the second day after modeling, the injected ankle joint was dropsical; some rats also lifted the left hind paw while moving.

### 2.3. Disposal for Each Group

50 *μ*L of histamine (100 *μ*g/mL in normal saline vehicle, histamine from Sigma-Aldrich) was injected at Zusanli (ST-36) (half under the skin, half in the muscle) in the His group. The Acu group was treated with acupuncture (described below). Prior to histamine injection (5 min), 20 *μ*L of a mast cell stabilizer, disodium cromolyn (0.02 g/mL in normal saline vehicle, disodium cromolyn from Sigma-Aldrich), was injected at the acupoint (half under the skin, half in the muscle) in the Cro + His group as a control for the other mast cell-degranulating substance. 50 *μ*L of the histamine H1 receptor antagonist clemastine (0.01 *μ*g/mL in normal saline vehicle, clemastine from Ingtech) was injected 5 min before acupuncture in the Cle + Acu group to study histamine function during animal acupuncture. To determine the efficiency of clemastine, a Cle group with a clemastine injection and a Cle + His group with an additional histamine injection were studied. To determine the efficiency of disodium cromolyn, a Cro + Acu group, which received a disodium cromolyn injection 5 min before acupuncture, was studied. The other groups included the Control group without modeling, the model group without treatment, and the NS group, which received an injection of 50 *μ*L of normal saline solution.

### 2.4. Nociceptive Testing Model

The thermal-induced paw withdraw test was used to assess analgesic responses. An analgesia meter (IITC, Life Sciences, Woodland Hills, C.A., U.S.) was used to apply heat stimulation. Each time, rats were acclimated to the test chamber for 30–40 min prior to testing. The triangle area of the underside of the left ankle joint was stimulated with 30% of maximum light strength. The room temperature was controlled at 24 ± 2°C. A 20 sec cutoff maximum was programmed into the timer to prevent tissue damage. We tested three times successively to obtain an average PT (10 min intervals were allowed between each test). 

Three PTs were tested in this study. Before modeling (BM), the PT was obtained before anesthesia for modeling; after modeling (AM), the PT was obtained on the second day after modeling. The treatment for each group was performed 1 hour after the AM test, and after treatment (AT), the PT was obtained 20 min after treatment. For the control and model groups, the AM and AT values were obtained according to the duration of the other groups.

### 2.5. Acupuncture Stimulation

Since Zusanli (ST-36) is a popular acupoint for analgesia studies in animal experiments as well as for clinical treatment, it was selected as the acupoint for our experiments. Sterilized, stainless steel acupuncture needles (0.25 mm in diameter, 1 inch in length, Suzhou Kangnian Medical Devices Co., Ltd., Suzhou, China) were inserted into ST-36 at the left hind leg, located 5 mm lateral and distal to the anterior tubercle of the tibia. The perpendicular needling depth was approximately 5 mm, and we alternately applied the lift-thrusting and twisting manipulation for 30 sec with 30 sec intervals. The acupuncture was performed for 30 min. 

### 2.6. Specimen Preparations and Microscopic Examination

**Figure 1:**
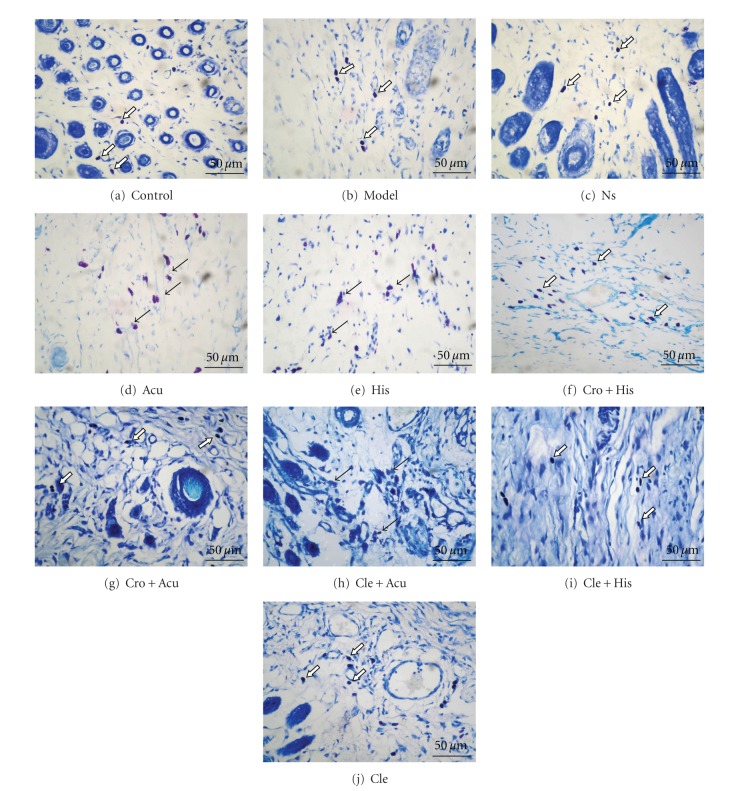
Mast cells in the skin and connective tissue near the acupoint area. (a) Control group; (b) Model group; (c) NS group; (d) Acu group; (e) His group; (f) Cro + His group; (g) Cro + Acu group; (h) Cle + Acu group; (i) Cle + His group; (j) Cle group. All pictures were taken at the dermis of ST-36, for groups receiving acupuncture (d), (g), (h) the textures are disoriented, for other group fibers are orderly placed. Blank arrows indicate mast cells in a stable state, and black arrows indicate degranulated mast cells (TB staining 400x).

Tissue samples from acupoints and nearby sham points were collected after decapitation of the animals under narcosis (10% chloral hydrate 0.4 mL/100 g i.p.). We took the upper part of tissue from ST-36. After cutting, the final size of the tissue sample with skin and muscle was 5 × 5 × 5 mm^3^. Sequential paraffin slices with 4-*μ*m thickness were made after 48 h of fixation at 4°C in fixing solution (10% formalin). The sections were longitudinal to the skin and the muscle tissue. The sample was stained with 0.5% toluidine blue. Mast cells could with more than three granules outside of the cell membrane or with empty cavities in the cytoplasm were considered degranulated (Figure 1). The numbers of mast cells per sample were counted and then averaged. Degranulation ratios (DR) which stands for the ratios of degranulated to total mast cells were calculated. Representative photomicrographs were taken at 400x magnification for morphological evaluation.

### 2.7. Data Analysis

Data were analyzed using SPSS 10.0. PT data were compared using a multivariable analysis. The mast cell degranulation rate was determined using a one-way ANOVA.

## 3. Results

### 3.1. Validity for Methods

The BM values for all the groups were not significantly different from each other, which indicated the stability of the animals' sensitivity to the thermal stimulation. After modeling, all of the other groups exhibited significant different sensitivity (versus control, Table 1), indicating hyperalgesia due to adjuvant-induced arthritis. 

The clemastine injection had no influence on the PT or DR (Cle versus NS, Table 1; Figure 1(j)). Disodium cromolyn successfully suppressed acupuncture-induced analgesia and reduced DR (Cro + Acu versus Acu, Table 1; Figures 1(d) and 1(g)). Both of these chemicals presented no effects other than their desired functions.

### 3.2. Acupuncture Analgesia Is Histaminergic

After the acupuncture treatment, both the PT and DR increased compared with the model group, which indicates that the analgesic effect was related to mast cell activation. In the Cle + Acu group, pretreatment with clemastine suppressed acupuncture-induced analgesia (versus Acu, Table 1). However, the mast cells were still activated by acupuncture-induced mechanical stimulations (versus Cle, Table 1; Figures 1(h) and 1(j)), which indicates that the acupuncture-induced analgesia is histaminergic in this case. 

### 3.3. Histamine Injection in the Acupoint Induces Analgesic Effects

Histamine injection had an analgesic effect (versus model, Table 1), while clemastine pretreatment suppressed this effect (versus His, Table 1). However, disodium cromolyn pretreatment had no significant effects (versus His, Table 1), and mast cells were stabilized without a significant increase in DR (versus His, Table 1; Figures 1(f) and 1(i)). Combined with the fact that there is no histamine H1 receptors on mast cells, the activation of mast cells in His group might be caused by substance P released from those afferents expressing histamine H1 receptors [3].

## 4. Discussion

In the acupoint, there are neural targets, including A*δ* and C-type fiber, which respond to manual acupuncture by inducing the central release of morphine peptide and thus the analgesic effect [5]. However, in clinical practice, the so-called “De-Qi” sensation determines the acupuncture's analgesic effects, which indicates the difference between acupuncture and nociceptive stimuli [6]. This kind of difference among acupuncture techniques might be caused by the mechanical activation of mast cells in the acupoint.

**Figure 2:**
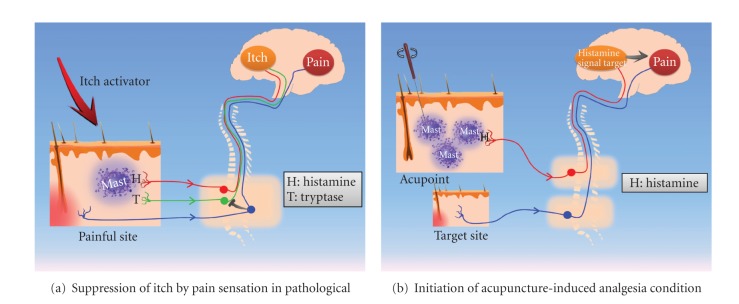
Interaction of histamine signal and pain sensation in case of itch suppression by pain and acupuncture analgesia. (a) In a pathological condition, pain suppresses the itch sensation. Mast cells are involved in the itch sensation in two possible ways: the activation of histamine receptors in a histamine-dependent fiber (red line) and the activation of PAR-2 by tryptase (green line). Both of these forms of activation are suppressed by the activation of pain (blue line) at the spinal level. (b) In the case of acupuncture, mast cells are activated by the mechanical force through the manipulation of the needle. The histamine release activated the histamine-dependent fiber through H1 receptors. Since the acupoint is away from the pain site, it is not interrupted by the activation of the pain sensation but activates the histamine target in the brain and initiates acupuncture analgesia.

The activation of mast cells is often related to the itch sensation, and this activation can be either histaminergic or nonhistaminergic (see Figure 2). A histamine-dependent itch (or pruritus) is a common itch sensation [7]. It is characterized by the triad effects of histamine in the skin, including flare, wheal, and itch. In the skin, histamine is synthesized in the Golgi apparatus of basophils and mast cells and is stored in granules inside of these cells. Mast cells in the skin can be activated by IgE, neurotransmitters, endocrines, or mechanical forces [8] and expel the granules, releasing histamine into the local environment [9]. Histamine-independent itch was first reported in 1953; papain and cowhage spicules were shown to induce the itch sensation [10]. The papain and cowhage spicules both activate polymodal C-fibers, which are in charge of pain sensation under mechanical and thermal stimuli as well [11]. The receptor target in this case is likely to be proteinase-activated receptor 2 (PAR2) [12], which can be activated by mast cell tryptase released from mast cells in both rat and human skin [13, 14].

In our study, we used the histamine H1 receptor antagonist clemastine to test the histamine dependence of acupuncture analgesia. We found that pretreatment with clemastine at the acupoint can suppress the analgesic effect of acupuncture, but it has no effect on the degranulation induced by acupuncture. The activation of neural regulation in the acupoint is histaminergic. 

Since the 1990s, some important discoveries regarding histamine-dependent itch had been made: Schemlz found a distinct subgroup of C-fibers that are preferentially excited by histamine [15]; Andrew and Craig found histamine-sensitive central projection neurons [16]. Both these authors suggested a histaminergic itch sensation pathway separate from that of pain. This hypothesis is also supported by the modulation of itch by pain in both direction [17] and displacement of pain and itch in pathological conditions [18, 19]. On human subject pain could markedly suppress itch in a range about 10 cm [20]. Histamine injection has direct effects on pain that cause dysesthesias around the site [21].

Considering these facts, in the present study, we tested histamine administration at the acupoint. We found that histamine injection in the acupoint provoked analgesia in a different segment. This effect was accompanied by mast cell degranulation, which might be the result of histamine-induced axon reflex. However, with disodium cromolyn pretreatment, this analgesic effect was not suppressed, which indicates that histamine plays a key role in the activation of the analgesic effect.

One unique characteristic of acupuncture remains: although there are about 360 acupoints in the human body, in practice, there is a preference for sites away from the target site. In traditional Chinese medicine, this preference is explained by the concept of balance. However, to date, not many efforts have been made in scientific acupuncture research on this topic. According to our findings, we believe, at least in the case of acupuncture analgesia, that this kind of preference might be caused by histamine-dependent initiation in the acupoint. As shown in Figure 2, in a pathological condition, at the painful site, both kinds of itch sensation will be suppressed by the activation of pain (blue line) on the spinal level. In this case, the histamine released from mast cells cannot generate activation in the central nervous system.  Figure 2(b) shows that in the case of acupuncture, mast cells are activated by mechanical force through the manipulation of needles. The histamine release activates the histamine-dependent fiber through H1 receptor, and since the acupoint is away from the pain site, it is not interrupted by pain sensation and activates the histamine-related center in brain, which might be responsible for acupuncture analgesia.

Clinical research of the past 40 years has demonstrated the effectiveness of acupuncture for relieving pain [22]. Studies on the central mechanism of acupuncture analgesia have been gaining attention for a long time [23]. However, because of the lack of knowledge about brain function and the mechanism of interactions between different sensations, the central mechanism of acupuncture's immediate effect had not previously been related to any physiological mechanism.

## 5. Conclusion

In our research, we found that during acupuncture, mast cells in the acupoint are activated and degranulated by the mechanical stimuli, and they can release histamine into the acupoint through the H1 receptor. The histamine modulates the microenvironment in the acupoint, generating an upstream signal and modulating pain sensation in the central nervous system. Moreover, the histamine dependence of the acupuncture analgesia indicates that acupuncture in the acupoint close to the target site is less effective because of the interruption of pathological pain in the target site. This finding reveals strategic differences between acupuncture analgesia and conventional pain regulation.

## Figures and Tables

**Table 1:** Comparison of PT and degranulation ratios of MCs near ST-36 among different groups.

Groups	*N *	Pain thresholds ($\overset{-}{x}\pm \text{s.e.}$, s)			Degranulation ratios ($\overset{-}{x}\pm \text{s.e.}$, %)
		Before model	After model	After treatment
Control	12	9.04 ± 0.20	9.38 ± 0.19	9.27 ± 0.17	33.59 ± 0.72
Model	12	8.90 ± 0.40	6.48 ± 0.28^#^	6.58 ± 0.35^*▵*^	39.71 ± 2.09
NS	11	9.23 ± 0.31	6.10 ± 0.33^#^	6.68 ± 0.33^*▵*^	37.72 ± 2.33
Acu	12	9.52 ± 0.18	6.58 ± 0.17^#^	8.77 ± 0.26*	57.61 ± 1.42*
His	12	9.21 ± 0.20	6.27 ± 0.22^#^	8.50 ± 0.28*	57.03 ± 2.95^∗▲^
Cro + His	13	9.21 ± 0.12	6.41 ± 0.19^#^	7.86 ± 0.30	25.40 ± 1.80^†^
Cro + Acu	12	8.91 ± 0.18	6.51 ± 0.19^#^	6.40 ± 0.36^‡^	36.03 ± 2.28^‡^
Cle + Acu	12	9.56 ± 0.32	6.90 ± 0.21^#^	6.54 ± 0.26^‡^	51.54 ± 2.32
Cle + His	12	9.36 ± 0.17	6.66 ± 0.18^#^	5.85 ± 0.28^†^	37.13 ± 1.90
Cle	22	9.56 ± 0.16	6.70 ± 0.21^#^	7.41 ± 0.2	32.24 ± 1.40

^#^ *P* < 0.01 versus control; *P < 0.01 versus model; ^*▵*^ *P* < 0.01 versus control; ^†^ *P* < 0.01 versus His; ^‡^ *P* < 0.01 versus Acu; ^▲^ *P* < 0.01 versus NS.

Data of control, model, NS, Acu, His groups were from earlier publication [4].
